# Synthesis, Anti-Breast Cancer Activity, and Molecular Docking Study of a New Group of Acetylenic Quinolinesulfonamide Derivatives

**DOI:** 10.3390/molecules22020300

**Published:** 2017-02-16

**Authors:** Krzysztof Marciniec, Bartosz Pawełczak, Małgorzata Latocha, Leszek Skrzypek, Małgorzata Maciążek-Jurczyk, Stanisław Boryczka

**Affiliations:** 1 Department of Organic Chemistry, School of Pharmacy with the Division of Laboratory Medicine in Sosnowiec, Medical University of Silesia in Katowice, Jagiellońska 4, 41-200 Sosnowiec, Poland; skrzypek@sum.edu.pl (L.S.); boryczka@sum.edu.pl (S.B.); 2 Department of Physical Pharmacy, School of Pharmacy with the Division of Laboratory Medicine in Sosnowiec, Medical University of Silesia in Katowice, Jagiellońska 4, 41-200 Sosnowiec, Poland; bpawelczak@sum.edu.pl (B.P.); mmaciazek@sum.edu.pl (M.M.-J.); 3 Department of Cell Biology, School of Pharmacy with the Division of Laboratory Medicine in Sosnowiec, Medical University of Silesia in Katowice, Jedności 8, 41-200 Sosnowiec, Poland; mlatocha@sum.edu.pl

**Keywords:** anticancer, quinolinesulfonamides, alkynes, cytochrome P450, virtual screening

## Abstract

In this study, a series of regioisomeric acetylenic sulfamoylquinolines are designed, synthesized, and tested in vitro for their antiproliferative activity against three human breast cacer cell lines (T47D, MCF-7, and MDA-MB-231) and a human normal fibroblast (HFF-1) by 4-[3-(4-iodophenyl)-2-(4-nitrophenyl)-2*H*-5-tetrazolio]-1,3-benzene disulfonate (WST-1) assay. The antiproliferative activity of the tested acetylenic quinolinesulfonamides is comparable to that of cisplatin. The bioassay results demonstrate that most of the tested compounds show potent antitumor activities, and that some compounds exhibit better effects than the positive control cisplatin against various cancer cell lines. Among these compounds, 4-(3-propynylthio)-7-[*N*-methyl-*N*-(3-propynyl)sulfamoyl]quinoline shows significant antiprolierative activity against T47D cells with IC_50_ values of 0.07 µM. In addition, 2-(3-Propynylthio)-6-[*N*-methyl-*N*-(3-propynyl)sulfa-moyl]quinoline and 2-(3-propynylseleno)-6-[*N*-methyl-*N*-(3-propynyl)sulfamoyl]quinoline display highly effective atitumor activity against MDA-MB-231 cells, with IC_50_ values of 0.09 and 0.50 µM, respectively. Furthermore, most of the tested compounds show a weak cytotoxic effect against the normal HFF-1 cell line. Additionally, in order to suggest a mechanism of action for their activity, all compounds are docked into the binding site of two human cytochrome P450 (CYP) isoenzymes. These data indicate that some of the title compounds display significant cytotoxic activity, possibly targeting the CYPs pathways.

## 1. Introduction

Interest in cancer has grown during the past century as infectious diseases have increasingly been controlled as a result of improved sanitation, vaccination, and antibiotics. Colon, stomach, liver, lung, and breast cancers are the most common causes of cancer death every year. Currently, breast cancer comprises 12% of all cancers in women worldwide. Moreover, the incidence of cancer continues to rise, and in many cases the applied treatment is ineffective [1,2]. Thus, many research studies are being performed, with the goal of identifying new medicines and treatment options.

The quinoline nucleus occurs in many classes of biologically active compound [3,4,5,6,7,8,9,10]. One of these classes is the quinolinesulfonamides, which have been studied as anticancer agents [7]. Sufamoylquinolines have displayed anti-HIV, neuroprotective, and antiviral properties [8], as well as antidepressant activity [9]. Quinolinesulfonamides have also inhibited carbonic anhydrases (CAs, EC 4.2.1.1) and have been associated with different human diseases (e.g., glaucoma and obesity) [10].

The interest in acetylenic compounds, especially terminal acetylenes, is continuously increasing because the introduction of the alkynyl group may significantly modify their chemical, physical, and biological properties. Synthetic methods for the preparation of this class of compounds are of inteest, especially with regard to the synthesis of biologically active enediyne and dienediyne antitumor antibiotics or similar molecular models [11,12,13,14,15]. The synthesis and some molecular properties of anticancer-active acetylenic sulfanylquinolines have been described previously [6,16,17,18,19,20,21]. Some of these compounds exhibit significant in vitro antiproliferative activity against human cancer cell lines, with the possible mechanism of action being cytochrome P450 (CYP) inhibitors.

The CYP family plays a crucial role in the detoxification and bioactivation of xenobiotics such as benzo[α]pyrene, and physiologically relevant compounds such as estradiol [22]. Aromatic compounds containing the acetylene functional group have long been recognized as mechanism-based inhibitors of CYPs. Various acetylenic aromatic compounds have been prepared, and their inhibitory activities evaluated on human cytochrome P450 1A1 and P450 1B1 isoenzymes (CYP1A1 and CYP1B1 respectively) [23]. CYP1A1 shares approximately 40% identity with CYP1B1. CYP1 mebers exhibit differences in tissue distribution—CYP1A1 and CYP1B1 are primarily localized in extrahepatic tissues. High expression levels of CYP1B1 have been found in steroidogenic tissues, icluding mammary tissues [23,24]. During the hydroxylation of estradiol, CYP1A1 mainly produces the 2-hydroxylation metabolite, whereas CYP1B1 is more effective in the production of the procacinogenic estrogen metabolite 4-hydroxyestradiol [25]. The importance of estrogens in the etiology of breast cancer is widely recognized [26,27,28]. Estrogens have been implicated in the initiation and promotion stages of breast cancer, and lifetime estrogen exposure is a major risk factor for breast cancer development. Estrogens exert their carcinogenic mechanism as well as their genotoxic mtabolites [29]. As a result, in 2001 steroidal estrogens were added to the list of known human cacinogens [30].

The expression of the CYP1 family in malignant tumors has important consequences for the treatment of cancer. The expression of CYP1, especially extrahepatic CYPs 1A1 and 1B1 in tumor cells provides a molecular target for the development of new anticancer drugs, especially in the case of hormone-dependent cancer diseases. The generally accepted mechanism of inhibition by the acetylenes involves two possible pathways. The first pathway involves the oxidation of the internal carbon of the triple bond, which leads to heme destruction and enzyme deactivation through the formation of an Fe–O–CR=C(•/+)–Heme complex where a radical or positive charge is localized on the terminal carbon. The second pathway involves the oxidation of the terminal carbon of the triple bond, which results in the formation of a reactive ketene intermediate. This ketene intermediate can covalently bind to a nucleophilic amino acid residue in the enzyme’s active site, leading to irrversible inhibition without destruction of the heme [31].

Against this background and in the continuation of efforts to synthesize potentially useful quinoline-based molecules [6,16,17,18,19,20,21], an efficient method has developed for the synthesis of novel acetylenic sulfamoylquinolines. The antiproliferative activity against human breast cancer cell lines, including MCF-7, T47D, and MDA-MB-231 as well as normal fibroblasts (HFF-1) using the WST-1 assay, is reported here. In order to suggest a mechanism of action for their activity, molecular docking on the active site of human CYP1A1 and CYP1B1 has been performed for all tested compounds.

## 2. Results and Discussion

### 2.1. Chemistry

**Scheme 1:**
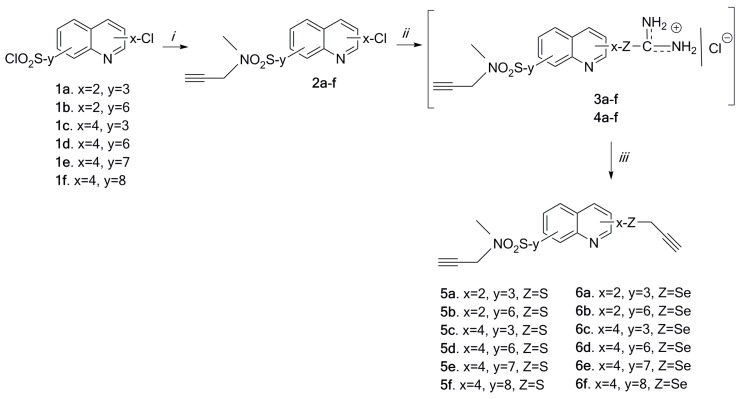
Reagents and conditions: *i*: (CH_3_CH_2_)_3_N, NH(CH_3_)CH_2_C≡CH, CHCl_3_, 0–5 °C, 3 h; *ii*: SC(NH_2_)_2_ or SeC(NH_2_)_2_, CH_3_CH_2_OH, r.t., 8 h; *iii*: 5% NaOH_aq_, CH≡CCH_2_Br, r.t., 45 min.

The starting compounds, chloroquinolinesulfonyl chlorides **1a**–**f**, were obtained from their respective dihalogenoquinolines, as described previously by [32,33]. A high yield of chlro-[*N*-methyl-*N*-(propynyl)sulfamoyl]quinolines **2a**–**f** was produced by amination of chloroquinolinesulfonyl chlorides **1a**–**f** with *N*-methylpropargylamine in chloroform with an excess of trethylamine (Scheme 1). Chloro-sulfamoylquinolines **2a**–**f** were converted into uronium salts **3a**–**f** and **4a**–**f** by nucleophilic displacement of the chlorine atom by thiourea or selenourea in ethanol. Hydrolysis of salts **3** and **4** and subsequent S- (or Se-) alkylation of sodium thiolates (or selenoates) with propargyl bromide resulted in the synthesis of compounds **5a**–**f** and **6a**–**f** (Scheme 1). The crude products were isolated from aqueous sodium hydroxide by filtration.

**Scheme 2:**
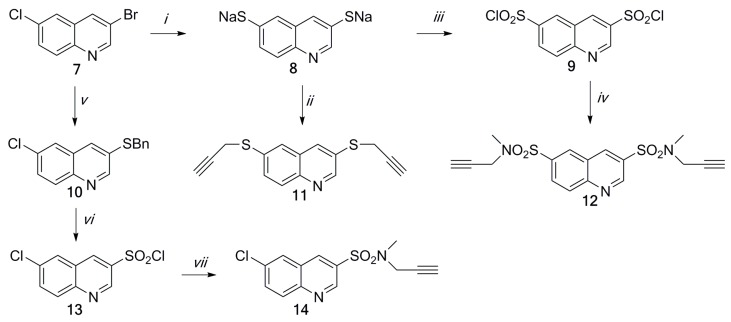
Reagents and conditions: *i*: CH_3_SNa, DMF, b.p. 6 h; *ii*: 5% NaOH_aq_, C≡CHCH_2_Br, r.t., 1 h; *iii*: HCl_conc_, 6% NaOCl_aq_, r.t., 45 min; *iv*: (CH_3_CH_2_)_3_N, NH(CH_3_)CH_2_C≡CH, CHCl_3_, 0–5 °C, 5 h; *v*: C_6_H_5_CH_2_SC(NH_2_)_2_^+^Cl^−^, NaOH, DMF, 80 °C, 7 h; *vi*: 10% HCl, CHCl_3_, 6 % NaOCl_aq_, 5–10 °C, 60 min; CH_3_CH_2_)_3_N, NH(CH_3_)CH_2_C≡CH, CHCl_3_, 0–5 °C, 3 h.

Dipropragylthioquinoline **11** was produced from 3-bromo-6-chloroquinoline **7** in a one-pot process performed with an excess of sodium methanethiolate (12 molar excess) (Scheme 2). This process proceeded stepwise by halogen substitution resulting in 3,6-dimethylthioquinoline (not shown in Scheme 2), which was then S-dealkylated to respective azinedithiolate **8**. This can be trapped by alkylation with propargyl bromide to produce dipropargylthioquinoline **11,** or oxidtively chlorinated to 3,6-dichlorosulfonylquinoline **9** as described previously [34].

The reaction of 3-bromo-6-chloroquinoline **7** with sodium phenylmethanethiolate (generated in situ from S-phenylmethylisothiouronium sulfate and sodium hydroxide) proceeded mainly by sustitution of the 3-bromine substituent to give the respective 6-chloro-3-phenylmethylsulfanylquinoline **10**. Phenylmethylsulfanylquinoline **10** was subjected to oxidative chlorination with a system of 6% sodium hypochlorite/10% hydrochloric gaseous chlorine, which proceeded by splitting of the benzyl group and oxidative chlorination of the 3-thioquinoline fragment to provide the expected 3-quinolinesulfonyl chloride **13**. Compounds **9** and **13** were then converted to the respective quinolinesulfonamides **12** and **14** by amination with *N*-methylpropargylamine with the presence of trimethylamine in chloroform with good yields (Scheme 2).

All of the final compounds were pure and stable, as characterized by proton nuclear magnetic resonance (^1^H-NMR), carbon nuclear magnetic resonance (^13^C-NMR), infrared spectroscopy (IR), and high-resolution mass spectrometry (HRMS). The ^1^H-NMR spectra of propargylsulfamoylquinolines **2**, **5**, **6**, **11**, **12**, and **14** show the typical proton signals for the propargyl group at 1.97–2.35 ppm (trplet) and 3.61–4.40 ppm (doublet) with a coupling constant of ^4^*J* = 2.7 Hz. In the ^13^C-NMR spectra of compounds **2**, **5**, **6**, **11**, **12**, and **14**, the signals at 10.7–40.6 ppm (-Se-CH_2_-, -S-CH_2_-, -N-CH_2_-), 70.8–75.0 ppm (-C≡), and 75.7–80.8 ppm (≡CH) are also attributed to the propargyl group. Moreover, in the ^1^H-NMR spectra of disubstituted quinolines **2**, **5**, **6**, **11**, **12**, and **14**, protons of the pyridine ring of the quinoline moiety formed an AX spin system, and protons of the benzene ring formed an AMX spin system. The assignments of both the ^1^H- and ^13^C-NMR spectra of the quinoline unit were based on the analysis of coupling patterns and the following two-dimensional nuclear magnetic resonance spectroscopy (2D NMR) experiments: correlation spectroscopy (COSY), heteronuclear multple-quantum correlation spectroscopy (HMQC), and heteronuclear multiple bond correlation spetroscopy (HMBC).

The IR spectra of compounds **2**, **5**, **6**, **11**, **12**, and **14** showed absorption bands at 3302–3211 cm^−1^, which are attributed to the ≡C–H stretching vibrations of the propargyl group. The C≡C stretching absorption for the propargyl group appeared at 2129–2010 cm^−1^. The asymmetric and symmetric SO_2_ stretching vibrations of compounds **2**, **5**, **6**, **12**, and **14** appeared as strong absorptions in the ranges 1365–1319 cm^−1^ and 1170–1132 cm^−1^, respectively.

The mass spectra of compounds **5**, **6**, **12**, and **14** exhibit signals corresponding to [M + Na]^+^. [M + 2 + Na]^+^ ion peaks, seen in compound **2**, due to the presence of chlorine or bromine atoms, which is consistent with the calculated value.

### 2.2. Biological Evaluation and SAR Analysis

Encouraged by promising results in the field of antiproliferative derivatives of acetylenic sulfanylquinoline derivatives [6,16,17,18,19,20,21], in this study the decision was made to combine the biological potentials of sulfamoylquinoline and alkynyl moieties, with the intention of obtaining compounds with better activity against breast cancer cell lines. The SAR analyses of the acetylenic sufamoylquinoline were based on the cell growth inhibition potencies on the MCF-7 (human adenocarcinoma), MDA-MB-231 and T-47D (human ductal carcinoma) cells using the WST-1 assay. The results from the in vitro cytotoxicity assay are expressed as the concentration of compound (µM) that inhibited the proliferation of 50% of the tumor cells, compared to the control untreated cells (IC_50_). Cisplatin was used as a positive control to induce cell death.

The SAR analysis started by varying chlorine substituent of the 2- or 4- position of quinoline moiety with the 3-, 6-, 7-, and 8-position being substituted by sulfamoyl group. We first synthesized eight compounds (**2a**–**f)**. Next, we introduced in positions 2- or 4- of the quinoline moiety the thipropargyl or the selenopropargyl group. A total of 21 compounds were synthesized. The results of the cytotoxicity studies of these compounds are summarized in Table 1. Most of the tested compounds exhibited higher anticancer activity than the reference drug, cisplatin. In this study, the obtained data indicate that the most active compounds against the MCF-7 cell line were in the folowing order: **5e** > **6f** > **2e** > **2c** > **12** > **11** > **6b**. As shown in Table 1, 4-propargylthio-7-[*N*-methyl-*N*-propargylsulfamoyl]quinoline **5e** had the highest antiproliferative activity (0.27 µM), which could be due to the presence of the thiopropargyl group in the 4- and sufamoyl group in the 7-quinoline positions. A structure–activity relationship was observed for the title compounds, and indicated that the replacement of a sulfur atom by a selenium atom retains activity against the MCF-7 cell line. In compound **6f**, which contains a selenopropargyl group at the 4-quinoline position, the IC_50_ values were comparable to those obtained for compound **5e**. Moreover, the MCF-7 cell line was found to be sensitive to 4-chloro-7-sulfamoyl- and 4-chloro-3-sulfamoyl-substituted quinolines **2e** and **2c** (0.38 and 0.50 µM, respectively). It is iportant to note that the MCF-7 cell line was found to be sensitive to 4-substituted quinolines. For the tested compounds, the ranked order of the anticancer activity against the T47D cell lines is as folows: **5e** > **5f** ≈ **2b** > **2c** > **12** > **6b**. As shown in Table 1, 4-propargylthio-7-sulfamoylquinoline **5e** and 4-propargylthio-8-sulfamoylquinoline **5f** had higher antiproliferative activity which could be due to the presence of the sulfamoyl group in the 7- or 8-quinoline position of the quinoline units. It is also noteworthy that compounds **5e** and **5f** showed higher cytotoxic activity than cisplatin in the tested cancer cell line, while their toxicity in normal human fibroblasts was low. However, the MCF-7 cell line was also found to be sensitive to 2,6-disubstituted quinoline **2b**.

The data indicated that the cytotoxic activity of the compounds against the MDA-MB-231 cell line was ranked in the following order: **6a** > **2b** > **2c** > **5b** > **6d** > **6b**. It is important to note that the MDA-MB-231 cell line was found to be highly sensitive to 2-substituted quinolines (2-propargylthio-3-sulfamoylquinoline **6a**, 2-chloro-6-sulfamoylquinoline **2b**, 2-propargylthio-6-sulfamoylquinoline **5b**, and 2-propargylseleno-6-sulfamoylquinoline **6b**).

It is noteworthy that compounds **2b**, **2c**, **5e**, **5f**, **5b**, **6b**, **11**, and **12** exhibited promising activity against the different cell lines used.

### 2.3. Docking Study

Molecular docking techniques were applied to investigate the binding mechanism for the series of acetylenic quinolinesulfonamides with human CYPs. Both CYP1A1 and CYP1B1 macromolecules are composed of 12 canonical α-helices (labeled from A to L), several short α-helices (labeled as A′, B′, etc.), and four canonical β-sheets (labeled from 1 to 4) with several β-strands [35,36]. Based on the atomic coordinates of CYP1A1 (PDB ID: 4I8V) and CYP1B1 (PDB ID: 3PM0) deposited in the Protein Data Bank (PDB) [37], two active site cavities were predicted (one for each protein). These cavities were located in the central part of the CYPs, deep inside the hydrophobic microenvironment of the four α-helices (B', F, G, I), the β-4 turn and the loop between the K helix and β-1 strand. Both cavities contained a heme molecule as the cofactor.

As a result of docking the computational models of quinolinesulfonamide derivatives to the active sites of CYP1A1 and CYP1B1, 42 possible ligand–protein complexes were obtained. All docked compounds were located in similar spaces inside the active sites, close to the heme-iron center. The superposition of studied compounds inside CYP1A1 and CYP1B1 (poses) were scored according to their intramolecular conformations (unfavorable ligand contributions) and binding interactions with amino acids (favorable ligand-protein contributions). Docking solutions with the lowest total score correspond to the highest binding affinities and present the most probable systems in vivo. The interaction energies between quinolinesulfonamides and CYPs are summarized in Table 2. The docking results clearly indicate that propargylthio- and propargylseleno-substituted quinlinesulfonamides have a higher binding affinity to CYP1A1 and CYP1B1 than their chlro-substituted derivatives. The data returned show that the top 30% of the scored poses are ranked in the following orders: **5b** > **11** > **6c** > **6d** > **6b** > **6f** > **12** (for CYP1A1) and **6d** > **6b** > **6f** > **12** > **5f** > **11** > **5b** (for CYP1B1). Steric interactions make the biggest contribution in the formation of all complexes. Hydrogen bonds occur very rarely and are only possible when compounds **6e**, **11** and **5e** bind to CYP1A1. In summary, the binding affinity of acetylenic quinolinesulfonamides to CYPs is positively related to their molecular weight and steric hindrance. Thiopropargyl or selenopropargyl compounds substituted in the positions: 3,2-, 3,4- and 4,8- of the quinoline moiety or its chloro derivtives should not form stable complexes with CYPs in vivo.

In order to find the best in silico orientation of docked compounds inside the CYP1A1 and CYP1B1 active sites to allow the inhibition of enzymes by ligands, the distances between the ligands’ functional groups and ferrous ion in heme molecules have been calculated (see Table S1 in the Supplementary Materials). Most of the docked derivatives are positioned with their sulfamoyl groups towards the catalytic heme-iron center, but three of them are directed towards the heme with their thiopropargyl (**5b**, **5e**, and **5f**) or selenopropargyl (**6a**) groups. The orientation of 3,6-di(3-propynylsulfanyl)quinoline (**11**) differs depending on the type of enzyme, in the active site of CYP1A1 ferrous ion is very close to the quinoline moiety of compound **11**, but in the active site of CYP1B1 its thiopropargyl group is oriented towards the heme.

**Figure 1:**
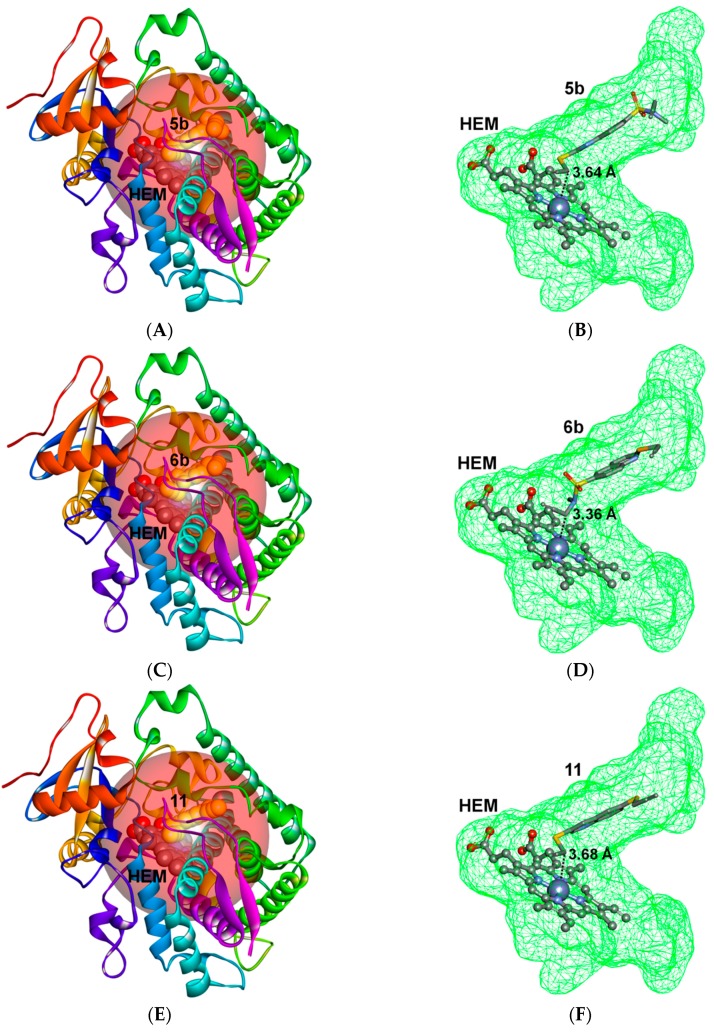
The tertiary structure of human CYP1A1 complexed with compounds **5b** (**A**), **6b** (**C**), and **11** (**E**), and a closer view of the orientation of compounds **5b** (**B**), **6b** (**D**), and **11** (**F**) inside the active site, near the heme–iron center. The protein’s backbone and active site are rendered as ribbon and green mesh, respectively.

**Figure 2:**
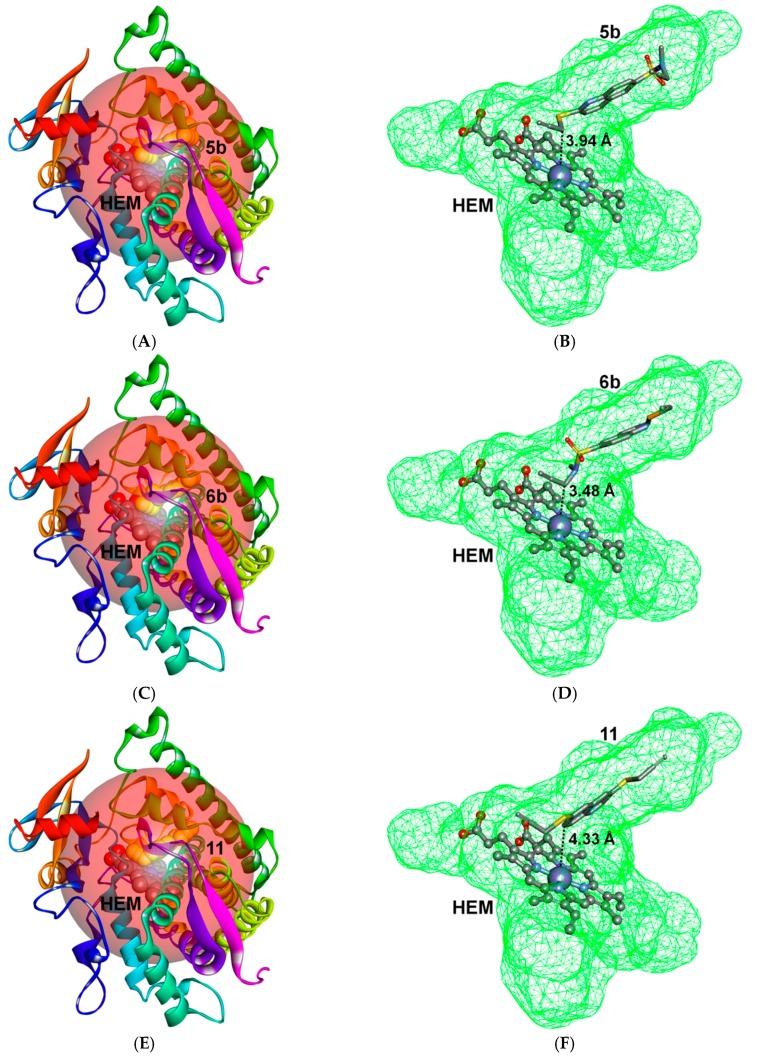
The tertiary structure of human CYP1B1 complexed with compounds **5b** (**A**), **6b** (**C**), and **11** (**E**) and a closer view of the orientation of compounds **5b** (**B**), **6b** (**D**), and **11** (**F**) inside the active site, near the heme–iron center. The protein’s backbone and active site are rendered as ribbon and green mesh, respectively.

For a detailed analysis of the molecular docking results, three compounds were selected that were docked close to the heme–iron center, and that have a high binding affinity to CYPs and very strong cytotoxic activity: **5b**, **6b**, and **11** (Figure 1 and Figure 2).

**Figure 3:**
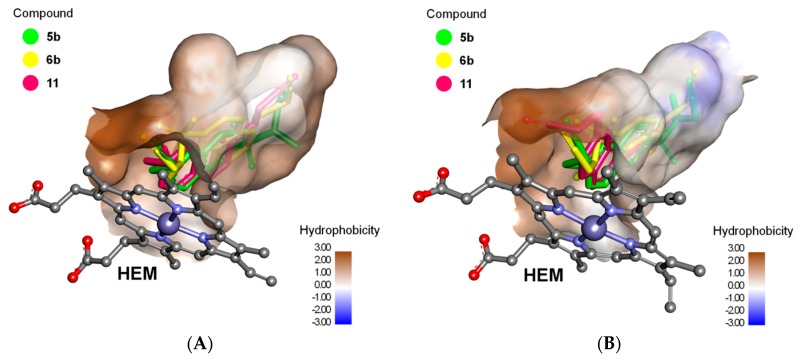
The superposition of compounds **5b**, **6b,** and **11** inside the active site’s cavity of human CYP1A1 (**A**) and CYP1B1 (**B**).

The chemistry of the active site's cavity in both enzymes (CYP1A1 and CYP1B1) shows that bounded acetylenic quinolinesulfonamides have deeply penetrated the strong hydrophobic matrix of these proteins. Figure 3 presents the geometry of the CYPs’ binding cavities, with compounds **5b**, **6b**, and **11** buried in the proteins’ hydrophobic environments, and the heme molecule as the cofactor.

The quinoline moiety of all docked acetylenic quinolinesulfonamides is stably anchored to the CYPs’ active sites via single or double π–π stacked and π–π T-shaped type interactions, with the benzyl side chain of phenylalanines in positions Phe123, Phe224, and Phe258 (CYP1A1) or Phe134, Phe231, and Phe268 (CYP1B1). The above-described highly conjugated system is additionally strengthened by single or double amide–π stacked interactions between the quinoline part and amide group, between residues of Gly316 and Ala317 (CYP1A1) or Gly329 and Ala330 (CYP1B1). Additionally, the quinoline moiety may form hydrophobic π–δ interactions with Phe123, Gly316, and Ala317 (CYP1A1) or Ala330 (CYP1B1). A number of π–alkyl interactions between quinoline rings and aliphatic side chains of Ile115, Leu312, Ala317 (CYP1A1), or Val126, Ala133, and Ala330 (CYP1B1) have also been observed. The differences in the mechanism of binding acetylenic quinlinesulfonamides to active sites of human CYPs are related to the positions of their sulfamoyl and/or thiopropargyl or selenopropargyl groups. Chlorine atoms in compounds from series 2 do not paticipate in the formation of complexes with CYPs, except for compounds **2a** and **2c**, which form a halogen non-bond interaction in the active site of CYP1B1 with negatively charged Asp333 residue. Detailed information about the non-bond interactions of acetylenic quinolinesulfonamides with the residues of CYP1A1 and CYP1B1 are summarized in Tables S2 and S3, respectively (see the Supplementary Materials).

**Figure 4:**
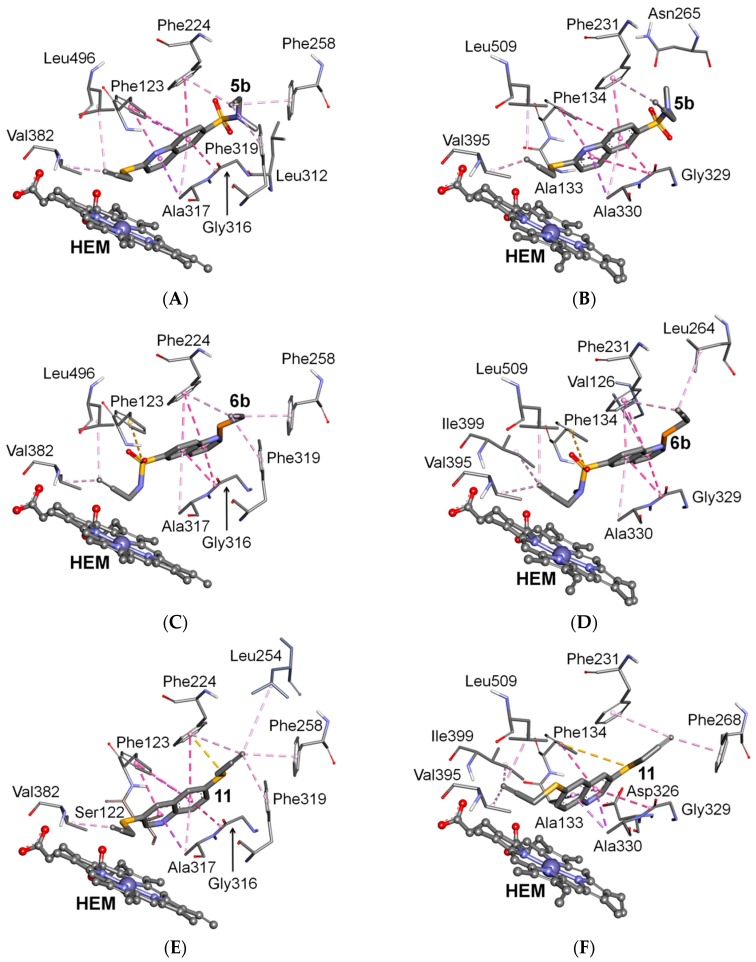
Binding site interactions between selected compounds and amino acid residues surrouning the active site of human CYPs. (**A**) **5b**-CYP1A1; (**B**) **5b**-CYP1B1; (**C**) **6b**-CYP1A1; (**D**) **6b**-CYP1B1; (**E**) **11**-CYP1A1; (**F**) **11**-CYP1B1. Hydrophobic (π–π, π–alkyl, alkyl–alkyl) and miscellaneous (sulfur) interactions are rendered as magenta and yellow dashed lines, respectively.

In Figure 4, molecules of **5b**, **6b**, and **11** are visualized in their optimized orientations inside CYP1A1 and CYP1B1, between the interacting amino acids and heme. The thiopropargyl group of molecule **5b** is oriented towards the ferrous ion of heme, inside the cavity of CYP1A1 or CYP1B1, and its superposition is stabilized by hydrophobic interactions with Val382 or Val395 and Leu496 or Leu509 aliphatic side chains (Figure 4A,B), respectively. In contrast to 5b, the sulfamoyl group of molecule **6b** is located inside the active sites of CYPs very close to ferrous ion, such that the pose provides the possibility of creating unique π–sulfur interactions with Phe123 (length 5.75 Å) of CYP1A1, and Phe134 (length 5.82 Å) of CYP1B1 (Figure 4C,D, respectively). The symmetric molecule of 3,6-dis(3-propynylthio)quinoline (**11**) docked to CYPs is oriented with the first thipropargyl group towards heme, and the second towards a highly conjugated system combined of three phenylalanines: Phe224, Phe258, and Phe319 in CYP1A1, or Phe134, Phe231, and Phe268 in CYP1B1 (Figure 4E,F, respectively).

**Figure 5:**
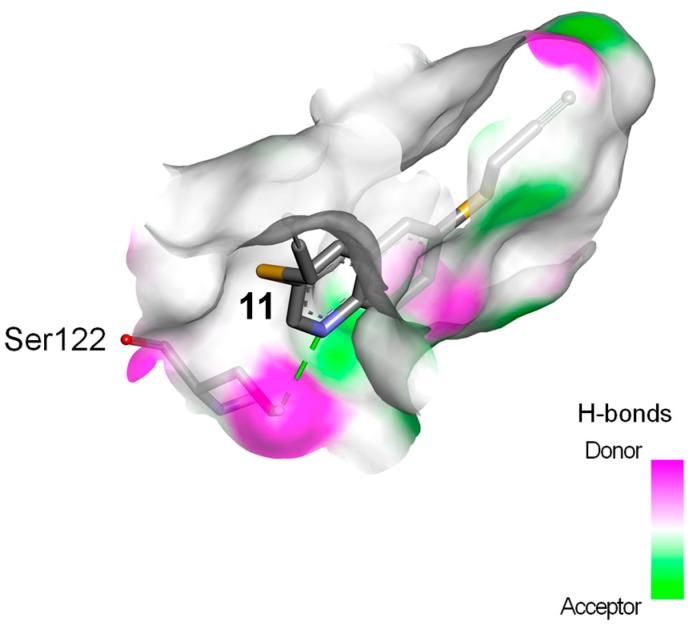
Conventional hydrogen bond (green and dashed line) formed between compound **11** and Ser122 residue in the active site of human CYP1A1. The active site’s cavity is visualized as a solvent and partially transparent surface.

Only one conventional hydrogen bond (length 2.56 Å) has a small contribution (score = **−**1.88 a.u.) in the formation of a complex between compound **11** (nitrogen atom acts as acceptor) and CYP1A1 (hydroxyl group of Ser 122 residue acts as a donor). Figure 5 shows the hydrogen bond between compound **11** and Ser 122 in the active site’s cavity of CYP1A1.

## 3. Materials and Methods

### 3.1. General Techniques 

Melting points were measured on an Electrothermal IA 9300 melting point apparatus. The ^1^H- and ^13^C-NMR spectra were determined using a Bruker Fourier 300 (Bruker, Billerica, MA, USA) in deuterated chloroform (CDCl_3_) or deuterated dimethyl sulfoxide (DMSO-*d*_6_); chemical shifts (δ) are reported in ppm and *J* values in Hz. The peak multiplicity is designated by a singlet (s), doublet (d), triplet (t), doublet of doublets (dd), doublet of triplets (dt), and multiplet (m). The HR MS analysis was performed on a Bruker Impact II instrument (Bruker). The IR spectra were recorded on an IRAffinity-1 Shimadzu spectrophotometer (Shimadzu Corporation, Kyoto, Japan). Thin layer chrmatography (TLC) was carried out on silica gel 60 254F plates (Merck, Darmstadt, Germany) using ethyl acetate as an eluent, and the spots were visualized by UV light (254 nm). All new compounds were purified by column chromatography. Silica gel 60 was used as a solid phase, and ethyl acetate was used as the eluent. The starting compounds, chloro-quinolinesulfochlorides **1a**–**f** and 3,6-dichlorosulfonylquinoline **9**, were obtained according to previously described methods [32,33,34].

### 3.2. General Procedure for the Synthesis of Chloro-[N-methyl-N-(3-propynyl)sulfamoyl]quinolines ***2a**–**f*** and ***14***

A solution of triethylamine (0.279 mL, 202 mg, 2 mmol) and *N*-methylpropargylamine (0.093 mL, 76 mg, 1.1 mmol) in 10 mL of chloroform was cooled to 0 °C and, while stirring, quinolinesulfonyl chloride **1a**–**f** or **13** (262 mg, 1 mmol) was then added. The stirring was continued at 0–5 °C for 3 h. Subsequently, the reaction mixture was washed with cold water (2 × 3 mL), dried with anhdrous sodium sulfate and concentrated under reduced pressure. The crude product was purified by column chromatography (ethyl acetate) to give pure products **2a**–**f** or **14**.

*2-Chloro-3-[N-methyl-N-(3-propynyl)sulfamoyl]quinoline* (**2a**). Yield: 89%, m.p. 110–111 °C. ^1^H-NMR (CDCl_3_, 300 MHz) δ: 2.20 (t, *J* = 2.7 Hz, 1H, CH), 3.08 (s, 3H, CH_3_), 4.22 (d, *J* = 2.7 Hz, 2H, CH_2_), 7.69 (ddd, *J* = 7.8 Hz, *J* = 7.6 Hz, *J* = 1.2 Hz, 1H, H-6), 7.89–7.99 (m, 2H, H-5 and H-7), 8.10 (dd, *J* = 7.6 Hz, *J* = 1.2 Hz, 1H, H-8), 8.98 (s, 1H, H-4). ^13^C-NMR (CDCl_3_, 75 MHz) δ: 34.7 (CH_3_), 39.7 (CH_2_CCH), 73.9 (CH_2_CCH), 76.8 (CH_2_CCH), 125.7 (C-4a), 128.4 (C-8), 128.5 (C-6) 128.9 (C-5), 130.8 (C-3), 133.5 (C-7), 142.9 (C-4), 145.3 (C-2), 148.4 (C-8a). IR (KBr, cm^−1^) ν_max_: 3292 (≡C-H), 3012 and 2966 (CH_2_, CH_3_), 2121 (C≡C), 1334 (S=O), 1153 (S=O). HRMS (ESI) *m*/*z*: C_13_H_11_ClN_2_NaO_2_S [M + Na]^+^, Calcd. 317.0127; Found 317.0119.

*2-Chloro-6-[N-methyl-N-(3-propynyl)sulfamoyl]quinoline* (**2b**). Yield: 95%, m.p. 140–141 °C. ^1^H-NMR (CDCl_3_, 300 MHz) δ: 1.97 (t, *J* = 2.4 Hz, 1H, CH), 2.92 (s, 3H, CH_3_), 4.14 (d, *J* = 2.4 Hz, 2H, CH_2_), 7.54 (d, *J* = 8.7 Hz, 1H, H-3), 8.08–8.17 (m, 2H, H-7 and H-8), 8.26 (d, *J* = 8.7 Hz, 1H, H-4), 8.40, (d, *J* = 1.5 Hz, 1H, H-5). ^13^C-NMR (CDCl_3_, 75 MHz) δ: 34.5 (CH_3_), 39.8 (CH_2_CCH), 74.4 (CH_2_CCH), 75.9 (CH_2_CCH), 124.1 (C-3), 125.8 (C-4a), 128.2 (C-7), 128.9 (C-5), 129.8 (C-8), 135.9 (C-6), 139.8 (C-4), 149.1 (C-8a), 153.7 (C-2). IR (KBr, cm^−1^) ν_max_: 3211 (≡C-H), 2924 and 2852 (CH_2_, CH_3_), 2113 (C≡C), 1325 (S=O), 1141 (S=O). HRMS (ESI) *m*/*z*: C_13_H_11_ClN_2_NaO_2_S [M + Na]^+^, Calcd. 317.0127; Found 317.0121.

*4-Chloro-3-[N-methyl-N-(3-propynyl)sulfamoyl]quinoline* (**2c**). Yield: 93%, m.p. 120–121 °C. ^1^H-NMR (CDCl_3_, 300 MHz) δ: 2.12 (t, *J* = 2.7 Hz, 1H, CH), 3.07 (s, 3H, CH_3_), 4.23 (d, *J* = 2.7 Hz, 2H, CH_2_), 7.77 (ddd, *J* = 7.9 Hz, *J* = 7.8 Hz, *J* = 1.2 Hz, 1H, H-6), 7.93 (ddd, *J* = 7.8 Hz, *J* = 7.6 Hz, *J* = 1.2 Hz, 1H, H-7), 8.20 (dd, *J* = 7.8 Hz, *J* = 1.2 Hz, 1H, H-8), 8.45 (dd, *J* = 7.9 Hz, *J* = 1.2 Hz, 1H, H-5), 9.39 (s, 1H, H-2). ^13^C-NMR (CDCl_3_, 75 MHz) δ: 34.6 (CH_3_), 39.6 (CH_2_CCH), 74.1 (CH_2_CCH), 76.5 (CH_2_CCH), 125.4 (C-5), 126.1 (C-4a), 129.0 (C-6), 129.9 (C-3), 130.0 (C-8), 132.9 (C-7), 142.9 (C-4), 149.4 (C-2), 149.9 (C-8a). IR (KBr, cm^−1^) ν_max_: 3265 (≡C-H), 2951 (CH_2_, CH_3_), 2118 (C≡C), 1334 (S=O), 1143 (S=O). HRMS (ESI) *m*/*z*: C_13_H_11_ClN_2_NaO_2_S [M + Na]^+^, Calcd. 317.0127; Found 317.0123.

*4-Chloro-6-[N-methyl-N-(3-propynyl)sulfamoyl]quinoline* (**2d**). Yield: 91%, m.p. 124–125 °C. ^1^H-NMR (CDCl_3_, 300 MHz) δ: 2.00 (t, *J* = 2.4 Hz, 1H, CH), 2.93 (s, 3H, CH_3_), 4.16 (d, *J* = 2.4 Hz, 2H, CH_2_), 7.64 (d, *J* = 4.8 Hz, 1H, H-3), 8.14 (dd, *J* = 9.0 Hz, *J* = 2.1 Hz, 1H, H-7), 8.28 (d, *J* = 9.0 Hz, 1H, H-8), 8.78 (d, *J* = 2.1 Hz, 1H, H-5), 8.93 (d, *J* = 4.8 Hz, 1H, H-2). ^13^C-NMR (CDCl_3_, 75 MHz) δ: 34.4 (CH_3_), 39.9 (CH_2_), 74.5 (CH_2_CCH), 75.9 (CH_2_CCH), 122.6 (C-3), 125.7 (C-5), 125.9 (C-4a), 128.2 (C-7), 130.9 (C-8), 136.8 (C-6), 144.4 (C-4), 149.9 (C-8a), 152.2 (C-2). IR (KBr, cm^−1^) ν_max_: 3267 (≡C-H), 3194 and 3086 (CH_2_, CH_3_), 2110 (C≡C), 1336 (S=O), 1155 (S=O). HRMS (ESI) *m*/*z*: C_13_H_11_ClN_2_NaO_2_S [M + Na]^+^, Calcd. 317.0127; Found 317.0125.

*4-Chloro-7-[N-methyl-N-(3-propynyl)sulfamoyl]quinoline* (**2e**). Yield: 94%, m.p. 119–120 °C. ^1^H-NMR (CDCl_3_, 300 MHz) δ: 2.01 (t, *J* = 2.7 Hz, 1H, CH), 2.93 (s, 3H, CH_3_), 4.15 (d, *J* = 2.7 Hz, 2H, CH_2_), 7.64 (d, *J* = 4.5 Hz, 1H, H-3), 8.02 (dd, *J* = 9.0 Hz, *J* = 1.5 Hz, 1H, H-6), 8.39 (d, *J* = 9.0 Hz, 1H, H-5), 8.64 (d, *J* = 1.5 Hz, 1H, H-8), 9.92 (d, *J* = 4.5 Hz, 1H, H-2). ^13^C-NMR (CDCl_3_, 75 MHz) δ: 34.5 (CH_3_), 39.9 (CH_2_CCH), 74.5 (CH_2_CCH), 75.9 (CH_2_CCH), 123.4 (C-3), 125.1 (C-6), 125.7 (C-5), 128.6 (C-4a), 130.6 (C-8), 139.2 (C-7), 142.8 (C-4), 148.2 (C-8a), 151.6 (C-2). IR (KBr, cm^−1^) ν_max_: 3282 (≡C-H), 3039 and 2935 (CH_2_, CH_3_), 2019 (C≡C), 1332 (S=O), 1161 (S=O). HRMS (ESI) *m*/*z*: C_13_H_11_ClN_2_NaO_2_S [M + Na]^+^, Calcd. 317.0127; Found 317.0128.

*4-Chloro-8-[N-methyl-N-(3-propynyl)sulfamoyl]quinoline* (**2f**). Yield: 90%, m.p. 130–133 °C. ^1^H-NMR (CDCl_3_, 300 MHz) δ: 2.03 (t, *J* = 2.4 Hz, 1H, CH), 3.00 (s, 3H, CH_3_), 4.39 (d, *J* = 2.4 Hz, 2H, CH_2_), 7.62 (d, *J* = 4.8 Hz, 1H, H-3), 7.74 (dd, *J* = 8.4 Hz, *J* = 7.5 Hz, 1H, H-6), 8.49 (dd, *J* = 8.4 Hz, *J* = 1.2 Hz, 1H, H-5), 8.56 (dd, *J* = 7.5 Hz, *J* = 1.2 Hz 1H, H-7), 8.93 (d, *J* = 4.8 Hz, 1H, H-2). ^13^C-NMR (CDCl_3_, 75 MHz) δ: 34.6 (CH_3_) 40.4 (CH_2_CCH), 72.7 (CH_2_CCH), 78.1 (CH_2_CCH), 122.3 (C-3), 126.5 (C-6), 127.3 (C-4a), 129.7 (C-5), 134.0 (C-7), 137.5 (C-8), 143.1 (C-4), 145.0 (C-8a), 150.6 (C-2). IR (KBr, cm^−1^) ν_max_: 3254 (≡C-H), 2943 (CH_2_, CH_3_), 2015 (C≡C), 1319 (S=O), 1132 (S=O). HRMS (ESI) *m*/*z*: C_13_H_11_ClN_2_NaO_2_S [M + Na]^+^, Calcd. 317.0127; Found 317.0131.

*6-Chloro-3-[N-methyl-N-(3-propynyl)sulfamoyl]quinoline* (**14**). Yield: 90%, m.p. 146–147 °C ^1^H-NMR (CDCl_3_, 300 MHz) δ: 1.98 (t, *J* = 2.7 Hz, 1H, CH), 2.94 (s, 3H, CH_3_), 4.19 (d, *J* = 2.7 Hz, 2H, CH_2_), 7.83 (dd, 1H, *J* = 9.0 Hz, *J* = 2.4 Hz, H-7), 7.96 (d, 1H, *J* = 2.1 Hz, H-5), 8.15 (d, 1H, *J* = 9.0 Hz, H-8), 8.60 (d, 1H, *J* = 2.1 Hz, H-4), 9.23 (d, 1H, *J* = 2.1 Hz, H-2). ^13^C-NMR (DMSO-*d_6_*, 75 MHz) δ: 34.5 (CH_3_), 39.9 (CH_2_CCH), 74.9 (CH_2_CCH), 75.7 (CH_2_CCH), 127.0 (C-6), 127.5 (C-5), 131.2 (C-8), 131.8 (C-3), 133.4 (C-7), 134.2 (C-4a), 136.3 (C-4), 147.5 (C-2), 147.6 (C-8a). IR (KBr, cm^−1^) ν_max_: 3302 (≡C-H), 3041 and 2974 (CH_2_, CH_3_), 2129 (C≡C), 1340 (S=O), 1155 (S=O). HRMS (ESI) *m*/*z*: C_13_H_11_ClN_2_NaO_2_S [M + Na]^+^, Calcd. 317.0127; Found 317.0125.

### 3.3. General Procedure for the Synthesis of Propynylthio- or Propynylseleno-[(N-methyl-N-(3-propynyl)sulfamoyl]quinolines ***5a**–**f*** and ***6a**–**f***

A mixture of the chloro-[(*N*-methyl-*N*-(3-propynyl)sulfamoyl[quinoline **2a**–**f** (147 mg, 0.5 mmol) and thiourea (42 mg 0.55 mmol) or selenourea (68 mg, 0.55 mmol) in ethanol (1 mL) was stirred at room temperature for 8 h under an argon atmosphere. The mixture was then transferred to cold 5% aqueous NaOH (10 mL), and propargyl bromide (0.042 mL, 65 mg, 0.55 mmol) was added dropwise to the aqueous layer. The mixture was stirred for 45 min, and the resultant solid was fitered off, washed with water, and air-dried to give crude products **5a**–**f** and **6a**–**f**. The crude product was purified by column chromatography (ethyl acetate) to give pure propargylthio- or propagylseleno-(*N*-methyl-*N*-(3-propynyl)-sulfamoylquinolines **5a**–**f** and **6a**–**f**.

*2-(3-Propynylthio)-3-[N-methyl-N-(3-propynyl)sulfamoyl]quinoline* (**5a**). Yield: 86%, m.p. 100–101 °C. ^1^H-NMR (CDCl_3_, 300 MHz) δ: 2.16–2.19 (m, 2H, NCH_2_CCH and SCH_2_CCH), 2.98 (s, 3H, CH_3_), 4.17 (d, *J* = 2.7 Hz, 2H, SeCH_2_CCH), 4.21 (d, *J* = 2.4 Hz, 2H, NCH_2_CCH), 7.59 (ddd, *J* = 7.8 Hz, *J* = 7.6 Hz, *J* = 1.5 Hz, 1H, H-6), 7.80–7.89 (m, 2H, H-5 and H-7), 8.02 (dd, *J* = 7.6 Hz, *J* = 1.5 Hz, 1H, H-8), 8.70 (s, 1H, H-4). ^13^C-NMR (CDCl_3_, 75 MHz) δ: 19.2 (SCH_2_CCH), 34.5 (CH_3_), 39.8 (NCH_2_CCH), 70.8 (SCH_2_CCH), 73.9 (NCH_2_CCH), 76.9 (NCH_2_CCH), 79.5 (SCH_2_CCH), 124.2 (C-4a), 126.9 (C-6), 128.0 (C-8), 128.9 (C-5), 128.9 (C-5), 129.7 (C-3), 132.9 (C-7), 140.3 (C-4), 148.4 (c-8a), 154.3 (C-2). IR (KBr, cm^−1^) ν_max_: 3271 (≡C-H), 3263 (≡C-H), 2951 and 2910 (CH_2_, CH_3_), 2115 (C≡C), 1323 (S=O), 1159 (S=O). HRMS (ESI) *m*/*z*: C_16_H_14_N_2_NaO_2_S_2_ [M + Na]^+^, Calcd. 353.0394; Found 353.0403.

*2-(3-Propynylthio)-6-[N-methyl-N-(3-propynyl)sulfamoyl]quinoline* (**5b**). Yield: 81%, m.p. 164–165 °C. ^1^H-NMR (CDCl_3_, 300 MHz) δ: 2.20 (t, *J* = 2.7 Hz, 1H, NCH_2_CCH), 2.23 (t, *J* = 2.7 Hz, 1H, SCH_2_CCH), 2.90 (s, 3H, CH_3_) 4.12 (d, *J* = 2.7 Hz, 2H, SCH_2_CCH), 4.18 (d, *J* = 2.7 Hz, 2H, NCH_2_CCH), 7.34 (d, *J* = 9.0 Hz, 1H, H-3), 8.00–8.10 (m, 3H, H-4, H-7 and H-8), 8.30 (d, 1H, *J* = 1.8 Hz, H-5). ^13^C-NMR (CDCl_3_, 75 MHz) δ: 18.2 (SCH_2_CCH), 34.4 (CH_3_), 39.8 (NCH_2_CCH), 70.9 (SCH_2_CCH), 74.3 (NCH_2_CCH), 76.1 (NCH_2_CCH), 79.4 (SCH_2_CCH), 121.9 (C-3), 125.0 (C-4a), 127.6 (C-7), 129.0 (C-5), 129.1 (C-8), 134.0 (C-6), 136.5 (C-4), 149.3 (C-8a), 161.2 (C-2). IR (KBr, cm^−1^) ν_max_: 3286 (≡C-H), 3261 (≡C-H), 2974 and 2945 (CH_2_, CH_3_), 2113 (C≡C), 1325 (S=O), 1155 (S=O). HRMS (ESI) *m*/*z*: C_16_H_14_N_2_NaO_2_S_2_ [M + Na]^+^, Calcd. 353.0394; Found 353.0399.

*4-(3-Propynylthio)-3-[N-methyl-N-(3-propynyl)sulfamoyl]quinoline* (**5c**). Yield: 81%, m.p. 101–102 °C. ^1^H-NMR (CDCl_3_, 300 MHz) δ: 2.14–2.16 (m, 2H, 2 xCH), 3.05 (s, 3H, CH_3_), 3.78 (d, *J* = 2.4 Hz, 2H, SCH_2_CCH), 4.29 (d, *J* = 1.8 Hz, 2H, NCH_2_CCH), 7.69 (ddd, *J* = 7.8 Hz, *J* = 7.6 Hz, *J* = 1.2 Hz, 1H, H-6), 7.90 (ddd, *J* = 7.8 Hz, *J* = 7.6 Hz, *J* = 1.2 Hz, 1H, H-7), 8.20 (dd, *J* = 7.8 Hz, *J* = 1.2 Hz, 1H, H-8), 8.82 (dd, *J* = 7.8 Hz, *J* = 1.2 Hz, 1H, H-5), 9.47 (s, 1H, H-2). ^13^C-NMR (CDCl_3_, 75 MHz) δ: 25.9 (SCH_2_CCH), 34.7 (CH_3_), 39.9 (NCH_2_CCH), 73.6 (SCH_2_CCH), 74.1 (NCH_2_CCH), 77.2 (NCH_2_CCH), 78.4 (SCH_2_CCH), 127.8 (C-5), 128.7 (C-6), 130.0 (C-4a), 130.2 (C-6), 132.5 (C-7), 136.8 (C-3), 144.0 (C-4), 149.1 (C-2), 140.7 (C-8a). IR (KBr, cm^−1^) ν_max_: 3294 (≡C-H), 3267 (≡C-H), 2968 and 2926 (CH_2_, CH_3_), 2013 (C≡C), 1330 (S=O), 1147 (S=O). HRMS (ESI) *m*/*z*: C_16_H_14_N_2_NaO_2_S_2_ [M + Na]^+^, Calcd. 353.0394; Found 353.0390.

*4-(3-Propynylthio)-6-[N-methyl-N-(3-propynyl)sulfamoyl]quinoline* (**5d**). Yield: 88%, m.p. 139–140 °C. ^1^H-NMR (CDCl_3_, 300 MHz) δ: 2.01 (t, *J* = 2.7 Hz, 1H, NCH_2_CCH), 2.35 (t, *J* = 2.4 Hz, 1H, SCH_2_CCH), 2.91 (s, 3H, -CH_3_), 3.90 (d, *J* = 2.4 Hz, 2H, SCH_2_CCH), 4.15 (d, *J* = 2.7 Hz, 2H, NCH_2_CCH), 7.51 (d, *J* = 4.8 Hz, 1H, H-3), 8.09 (dd, *J* = 9.0 Hz, *J* = 1.8 Hz, 1H, H-7), 8.24 (d, *J* = 9.0 Hz, 1H, H-8), 8.61 (d, *J* = 1.8 Hz, 1H, H-5), 8.89 (d, *J* = 4.8 Hz, 1H, H-2). ^13^C-NMR (CDCl_3_, 75 MHz) δ: 20.0 (SCH_2_CCH), 34.5 (CH_3_) 39.9 (NCH_2_CCH), 73.0 (SCH_2_CCH), 74.6 (NCH_2_CCH), 75.9 (NCH_2_CCH), 77.3 (SCH_2_CCH), 117.8 (C-3), 124.8 (C-5), 125.5 (C-4a), 127.8 (C-7), 130.7 (C-8), 135.4 (C-6), 148.0 (C-4), 148.8 (C-8a), 151.0 (C-2). IR (KBr, cm^−1^) ν_max_: 3292 (≡C-H), 3250 (≡C-H), 3012 and 2966 (CH_2_, CH_3_), 2121 (C≡C), 1334 (S=O), 1153 (S=O). HRMS (ESI) *m*/*z*: C_16_H_14_N_2_NaO_2_S_2_ [M + Na]^+^, Calcd. 353.0394; Found 353.0391.

*4-(3-Propynylthio)-7-[N-methyl-N-(3-propynyl)sulfamoyl]quinoline* (**5e**). Yield: 90%, m.p. 113–114 °C. ^1^H-NMR (CDCl_3_, 300 MHz) δ: 2.01 (t, *J* = 2.1 Hz, 1H, NCH_2_CCH), 2.34 (t, *J* = 2.4 Hz, 1H, SCH_2_CCH), 2.94 (s, 3H, CH_3_), 3.88 (d, *J* = 2.4 Hz, 2H, SCH_2_CCH), 4.13 (d, *J* = 2.1 Hz, 2H, NCH_2_CCH), 7.50 (d, *J* = 4.8 Hz, 1H, H-3), 7.92 (dd, *J* = 8.7 Hz, *J* = 1.2 Hz, 1H, H-6), 8.20 (d, *J* = 8.7 Hz, 1H, H-5), 8.60 (d, *J* = 1.2 Hz, 1H, H-8), 8.88 (d, *J* = 4.8 Hz, 1H, H-2). ^13^C-NMR (CDCl_3_, 75 MHz) δ: 19.8 (SCH_2_CCH), 34.5 (CH_3_), 39.9 (NCH_2_CCH), 72.8 (SCH_2_CCH), 74.4 (NCH_2_CCH), 75.9 (NCH_2_CCH), 76.6 (SCH_2_CCH), 118.4 (C-3), 124.0 (C-6), 124.7 (C-5), 128.4 (C-4a), 130.7 (C-8), 138.5 (C-7), 146.2 (C-4), 146.5 (C-8a), 150.9 (C-2). IR (KBr, cm^−1^) ν_max_: 3255 (≡C-H), 2935 (CH_2_, CH_3_), 2014 (C≡C), 1348 (S=O), 1159 (S=O). HRMS (ESI) *m*/*z*: C_16_H_14_N_2_NaO_2_S_2_ [M + Na]^+^, Calcd. 353.0394; Found 353.0391.

*4-(3-Propynylthio)-8-[N-methyl-N-(3-propynyl)sulfamoyl]quinoline* (**5f**). Yield: 93%, m.p. 119–120 °C. ^1^H-NMR (CDCl_3_, 300 MHz) δ: 2.06 (t, *J* = 2.1 Hz, 1H, NCH_2_CCH), 2.32 (t, *J* = 2.4 Hz, 1H, SCH_2_CCH), 2.98 (s, 3H, CH_3_), 3.86 (d, *J* = 2.4 Hz, 2H, SCH_2_CCH), 4.40 (d, *J* = 2.1 Hz, 2H, NCH_2_CCH), 7.47 (d, *J* = 4.8 Hz, 1H, H-3), 7.63 (dd, *J* = 8.4 Hz, *J* = 7.5 Hz, 1H, H-6), 8.31 (dd, *J* = 8.4 Hz, *J* = 1.2 Hz, 1H, H-5), 7.90 (dd, *J* = 7.5 Hz, *J* = 1.2 Hz 1H, H-7), 8.90 (d, *J* = 4.8 Hz, 1H, H-2). ^13^C-NMR (CDCl_3_, 75 MHz) δ: 19.9 (SCH_2_CCH), 34.6 (CH_3_) 40.6 (NCH_2_CCH), 72.6 (SCH_2_CCH), 72.7 (NCH_2_CCH), 77.6 (SCH_2_CCH), 78.3 (NCH_2_CCH), 117.5 (C-3), 125.3 (C-6), 127.0 (C-4a), 128.9 (C-5), 133.4 (C-7), 137.4 (C-8), 143.7 (C-8a), 146.4 (C-4), 149.9 (C-2). IR (KBr, cm^−1^) ν_max_: 3261 (≡C-H), 3253 (≡C-H), 2957 and 2922 (CH_2_, CH_3_), 2016 (C≡C), 1325 (S=O), 1132 (S=O). HRMS (ESI) *m*/*z*: C_16_H_14_N_2_NaO_2_S_2_ [M + Na]^+^, Calcd. 353.0394; Found 353.0394.

*2-(3-Propynylseleno)-3-[N-methyl-N-(3-propynyl)sulfamoyl]quinoline* (**6a**). Yield: 89%, m.p. 109–110 °C. ^1^H-NMR (CDCl_3_, 300 MHz) δ: 2.13 (t, *J* = 2.4 Hz, 1H, NCH_2_CCH), 2.19 (t, *J* = 2.7 Hz, 1H, SeCH_2_CCH), 2.96 (s, 3H, CH_3_), 4.05 (d, *J* = 2.7 Hz, 2H, SeCH_2_CCH), 4.21 (d, *J* = 2.4 Hz, 2H, NCH_2_CCH), 7.59 (ddd, *J* = 7.8 Hz, *J* = 7.6 Hz, *J* = 0.9 Hz, 1H, H-6), 7.81–7.90 (m, 2H, H-5 and H-7), 8.04 (dd, *J* = 7.6 Hz, *J* = 0.9 Hz, 1H, H-8), 8.64 (s, 1H, H-4). ^13^C-NMR (CDCl_3_, 75 MHz) δ: 11.7 (SeCH_2_CCH), 34.6 (CH_3_), 39.9 (NCH_2_CCH), 70.8 (SeCH_2_CCH), 74.2 (NCH_2_CCH), 77.2 (NCH_2_CCH), 80.8 (SeCH_2_CCH), 124.4 (C-4a), 127.1 (C-6), 128.3 (C-8), 128.9 (C-5), 131.0 (C-3), 132.9 (C-7), 139.6 (C-4), 149.0 (C-8a), 152.5 (C-2). IR (KBr, cm^−1^) ν_max_: 3275 (≡C-H), 2918 and 2852 (CH_2_, CH_3_), 2121 (C≡C), 1321 (S=O), 1155 (S=O). HRMS (ESI) *m*/*z*: C_16_H_1_N_2_NaO_2_SeS [M + Na]^+^, Calcd. 400.9839; Found 400.9841.

*2-(3-Propynylseleno)-6-[N-methyl-N-(3-propynyl)sulfamoyl]quinoline* (**6b**). Yield: 89%, m.p. 179–180 °C. ^1^H-NMR (CDCl_3_, 300 MHz) δ: 1.99 (t, *J* = 2.7 Hz, 1H, NCH_2_CCH), 2.25 (t, *J* = 2.7 Hz, 1H, SeCH_2_CCH), 2.90 (s, 3H, CH_3_), 4.09 (d, *J* = 2.7 Hz, 2H, SeCH_2_CCH), 4.12 (d, *J* = 2.7 Hz, 2H, NCH_2_CCH), 7.47 (d, *J* = 8.7 Hz, 1H, H-3), 7.99–8.04 (m, 2H, H-4 and H-7), 8.09 (d, *J* = 8.7 Hz, 1H, H-8), 8.31 (d, *J* = 1.8 Hz, 1H, H-5). ^13^C-NMR (CDCl_3_, 75 MHz) δ: 10.7 (SeCH_2_CCH), 34.4 (CH_3_), 39.9 (NCH_2_CCH), 71.2 (SeCH_2_CCH), 74.3 (NCH_2_CCH), 76.0 (NCH_2_CCH), 80.6 (SeCH_2_CCH), 124.2 (C-3), 125.5 (C-4a), 127.6 (C-7), 129.2 (C-5 and C-8), 134.3 (C-6), 136.2 (C-4), 149.9 (C-8a), 159.5 (C-2). IR (KBr, cm^−1^) ν_max_: 3284 (≡C-H), 3261 (≡C-H), 2960 and 2904 (CH_2_, CH_3_), 2113 (C≡C), 1325 (S=O), 1157 (S=O). HRMS (ESI) *m*/*z*: C_16_H_1_N_2_NaO_2_SeS [M + Na]^+^, Calcd. 400.9839; Found 400.9836.

*4-(3-Propynylseleno)-3-[N-methyl-N-(3-propynyl)sulfamoyl]quinoline* (**6c**). Yield: 85%, m.p. 122–123 °C. ^1^H-NMR (CDCl_3_, 300 MHz) δ: 2.15 (t, *J* = 2.7 Hz, 1H, NCH_2_CCH), 2.22 (t, *J* = 2.4 Hz, 1H, SeCH_2_CCH), 3.04 (s, 3H, CH_3_), 3.61 (d, *J* = 2.4 Hz, 2H, SeCH_2_CCH), 4.31 (d, *J* = 2.7 Hz, 2H, NCH_2_CCH), 7.73 (ddd, *J* = 7.9 Hz, *J* = 7.8 Hz, *J* = 1.2 Hz, 1H, H-6), 7.88 (ddd, *J* = 7.8 Hz, *J* = 7.6Hz, *J* = 1.2 Hz, 1H, H-7), 8.18 (dd, *J* = 8 Hz, *J* = 1.2 Hz, 1H, H-8), 8.68 (dd, *J* = 7.9 Hz, *J* = 1.2 Hz, 1H, H-5), 9.42 (s, 1H, H-2). ^13^C-NMR (CDCl_3_, 150 MHz) δ: 16.0 (SeCH_2_CCH), 34.6 (CH_3_), 40.0 (NCH_2_CCH), 73.5 (SeCH_2_CCH), 74.2 (NCH_2_CCH), 77.4 (NCH_2_CCH), 79.5 (SeCH_2_CCH), 128.8 (C-6), 129.7 (C-4a), 130.0 (C-5), 130.4 (C-8), 132.4 (C-7), 137.0 (C-3), 142.0 (C-4), 148.5 (C-2), 149.2 (C-8a). IR (KBr, cm^−1^) ν_max_: 3273 (≡C-H), 3055 and 2978 (CH_2_, CH_3_), 2123 (C≡C), 1342 (S=O), 1155 (S=O). HRMS (ESI) *m*/*z*: C_16_H_1_N_2_NaO_2_SeS [M + Na]^+^, Calcd. 400.9839; Found 400.9838.

*4-(3-Propynylseleno)-6-[N-methyl-N-(3-propynyl)sulfamoyl]quinoline* (**6d**). Yield: 91%, m.p. 139–140 °C. ^1^H-NMR (CDCl_3_, 300 MHz) δ: 2.02 (t, *J* = 2.4 Hz, 1H, NCH_2_CCH), 2.34 (t, *J* = 2.4 Hz, 1H, SeCH_2_CCH), 2.92 (s, 3H, CH_3_), 3.78 (d, *J* = 2.4 Hz, 2H, SeCH_2_CCH), 4.15 (d, *J* = 2.4 Hz, 2H, NCH_2_CCH), 7.75 (d, *J* = 4.8 Hz, 1H, H-3), 8.11 (dd, *J* = 9.0 Hz, *J* = 1.8 Hz, 1H, H-7), 8.32 (d, *J* = 9.0 Hz, 1H, H-8), 8.57 (d, *J* = 1.8 Hz, 1H, H-5), 8.85 (d, *J* = 4.8 Hz, 1H, H-2). ^13^C-NMR (CDCl_3_, 75 MHz) δ: 11.9 (SeCH_2_CCH), 34.5 (CH_3_), 39.9 (NCH_2_CCH), 73.3 (SeCH_2_CCH), 74.6 (NCH_2_CCH), 75.9 (NCH_2_CCH), 78.6 (SeCH_2_CCH), 123.3 (C-3), 126.9 (C-5), 127.7 (C-4a), 128.0 (C-7), 130.6 (C-8), 136.0 (C-6), 146.6 (C-4), 147.9 (C-8a), 150.6 (C-2). IR (KBr, cm^−1^) ν_max_: 3290 (≡C-H), 3248 (≡C-H), 3012 and 2968 (CH_2_, CH_3_), 2010 (C≡C), 1332 (S=O), 1153 (S=O). HRMS (ESI) *m*/*z*: C_16_H_1_N_2_NaO_2_SeS [M + Na]^+^, Calcd. 400.9839; Found 400.9838.

*4-(3-Propynylseleno)-7-[N-methyl-N-(3-propynyl)sulfamoyl]quinoline* (**6e**). Yield: 82%, m.p. 108–109 °C. ^1^H-NMR (CDCl_3_, 300 MHz) δ: 2.02 (t, *J* = 2.7 Hz, 1H, NCH_2_CCH), 2.32 (t, *J* = 2.7 Hz, 1H, SCH_2_CCH), 2.92 (s, 3H, CH_3_), 3.76 (d, *J* = 2.7 Hz, 2H, SCH_2_CCH), 4.13 (d, *J* =2.7 Hz, 2H, NCH_2_CCH), 7.73 (d, *J* = 4.8 Hz, 1H, H-3), 7.95 (dd, *J* = 9.0 Hz, *J* = 1.8 Hz, 1H, H-6), 8.15 (d, *J* = 9.0 Hz, 1H, H-5), 8.63 (d, *J* = 1.8 Hz, 1H, H-8), 8.84 (d, *J* =4.8 Hz, 1H, H-2). ^13^C-NMR (CDCl_3_, 75 MHz) δ: 11.6 (SeCH_2_CCH), 34.5 (CH_3_), 39.9 (NCH_2_CCH), 73.1 (SeCH_2_CCH), 74.5 (NCH_2_CCH), 76.0 (NCH_2_CCH), 78.7 (SeCH_2_CCH), 123.6 (C-3), 124.7 (C-6), 126.8 (C-5), 129.8 (C-8), 130.3 (C-4a), 139.3 (C-7), 145.3 (C-4), 145.5 (C-8a), 149.8 (C-2). IR (KBr, cm^−1^) ν_max_: 3296 (≡C-H), 3275 (≡C-H), 3074 and 2980 (CH_2_, CH_3_), 2015 (C≡C), 1342 (S=O), 1161 (S=O). HRMS (ESI) *m*/*z*: C_16_H_1_N_2_NaO_2_SeS [M + Na]^+^, Calcd. 400.9839; Found 400.9834.

*4-(3-Propynylseleno)-8-[N-methyl-N-(3-propynyl)sulfamoyl]quinoline* (**6f**). Yield: 89%, m.p. 111–115 °C. ^1^H-NMR (CDCl_3_, 300 MHz) δ: 2.05 (t, *J* = 2.1 Hz, 1H, NCH_2_CCH), 2.31 (t, *J* = 2.7 Hz, 1H, SeCH_2_CCH), 2.99 (s, 3H, CH_3_), 3.72 (d, *J* = 2.7 Hz, 2H, SeCH_2_CCH), 4.40 (d, *J* = 2.1 Hz, 2H, NCH_2_CCH), 7.63–7.69 (m, 2H, H-3 and H-6), 8.27 (dd, *J* = 8.4 Hz, *J* = 1.5 Hz, 1H, H-5), 8.52 (dd, *J* = 7.5 Hz, *J* = 1.2 Hz, 1H, H-7), 8.86 (d, *J* = 4.5 Hz, H-2). ^13^C-NMR (CDCl_3_, 75 MHz) δ: 19.9 (SCH_2_CCH), 34.6 (CH_3_) 40.6 (NCH_2_CCH), 72.6 (NCH_2_CCH), 72.9 (SeCH_2_CCH), 78.3 (NCH_2_CCH), 78.9 (SeCH_2_CCH), 123.0 (C-3), 125.6 (C-6), 129.0 (C-4a), 131.1 (C-5), 133.5 (C-7), 137.5 (C-8), 143.3 (C-4), 143.9 (C-8a), 149.9 (C-2). IR (KBr, cm^−1^) ν_max_: 3257 (≡C-H), 2957 and 2925 (CH_2_, CH_3_), 2015 (C≡C), 1325 (S=O), 1132 (S=O). HRMS (ESI) *m*/*z*: C_16_H_1_N_2_NaO_2_SeS M + Na]^+^, Calcd. 400.9839; Found 400.9833.

### 3.4. Procedure for the Synthesis of 3,6-Di(3-propynylsulfanyl)quinoline *(**11**)*

A mixture of 6-chloro-3-bromoquinoline (242 mg, 1 mmol), sodium methanethiolate (840 mg, 12 mmol), and dry DMF (10 mL) was boiled with stirring under argon atmosphere for 6 h. The mixture was then cooled to room temperature and poured into cold 8% aqueous NaOH (30 mL), and prpargyl bromide (0.126 mL, 195 mg, 1.65 mmol) was added dropwise with stirring. The stirring cotinued at room temperature for 1 h. The solid was filtered off, washed with water, and air-dried. The crude product was purified by column chromatography (ethyl acetate) to give pure 3,6-di(3-propynylsulfanyl)quinoline (**11**).

*3,6-Di(3-propynylthio)quinoline* (**11**). Yield: 91%, m.p. 97–98 °C. ^1^H-NMR (CDCl_3_, 300 MHz) δ: 2.27–2.30 (m, 2H, 2 × CH), 3.70 (d, *J* = 2.7 Hz, 2H, SCH_2_), 3.74 (d, *J* = 2.7 Hz, 2H, SCH_2_), 7.70 (dd, 1H, *J* = 8.7 Hz, *J* = 2.1 Hz, H-7), 7.78 (d, *J* = 2.1 Hz, H-5), 8.05 (d, 1H, *J* = 8.7 Hz, H-8), 8.18 (d, 1H, *J* = 1.8 Hz, H-4), 8.88 (d, 1H, *J* = 1.8 Hz, H-2). ^13^C-NMR (CDCl_3_, 75 MHz) δ: 21.9 (SCH_2_CCH), 22.7 (SCH_2_CCH), 72.1 (SCH_2_CCH), 72.8 (SCH_2_CCH), 78.8 (SCH_2_CCH), 79.1 (SCH_2_CCH), 125.8 (C-5), 128.5 (C-4a), 129.2 (C-8), 129.7 (C-3), 131.0 (C-7), 135.4 (C-6), 136.6 (C-4), 144.6 (C-8a), 150.9 (C-2). IR (KBr, cm^−1^) ν_max_: 3278 (≡C-H), 2926 and 2885 (CH_2_), 2110 (C≡C). HRMS (ESI) *m*/*z*: C_15_H_12_NS_2_ [M + H]^+^, Calcd. 270.0411; Found 270.0415.

### 3.5. Procedure for the Synthesis of 3,6-Di[N-methyl-N-(3-propynyl)sulfamoyl]quinoline *(**12**)*

A solution of triethylamine (0.558 mL, 404 mg, 4 mmol) and *N*-methylpropargylamine (0.186 mL, 152 mg, 2.2 mmol) in 20 mL of chloroform was cooled to 0 °C and then, with stirring, 3,6-dichlorosulfonylquinoline **9** (326 mg, 1 mmol) was added. The stirring was continued at 0–5 °C for 5 h. Then, the reaction mixture was washed with cold water (2 × 6 mL), dried with anhydrous sodium sulfate, and concentrated under reduced pressure. The crude product was purified by column chromatography (ethyl acetate) to give pure product **12**.

*3,6-Di[N-methyl-N-(3-propynyl)sulfamoyl]quinoline* (**12**). Yield: 96%, m.p. 138–139 °C. ^1^H-NMR (CDCl_3,_ 300 MHz) δ: 1.97–1.99 (m, 2H, 2 × CH), 2.95 (s, 3H, CH_3_), 2.97 (s, 3H, CH_3_), 4.18 (d, *J* = 2.4 Hz, 2H, CH_2_), 4.22 (d, *J* = 2.4 Hz, 2H, CH_2_), 8.23 (dd, 1H, *J* = 9.0 Hz, *J* = 2.1 Hz, H-7), 8.36 (d, 1H, *J* = 9.0 Hz, H-8), 8.55 (d, 1H, *J* = 2.1 Hz, H5), 8.82 (d, 1H, *J* = 2.1 Hz, H-4), 9.38 (d, 1H, *J* =2.1 Hz, H-2). ^13^C-NMR (CDCl_3_, 75 MHz) δ: 34.5 (2 × CH_3_), 39.9 (2 × CH_2_), 74.6 (CH_2_CCH), 75.0 (CH_2_CCH), 75.7 (CH_2_CCH), 75.8 (CH_2_CCH), 125.6 (C-4a), 129.8 (C-7), 130.2 (C-5), 130.7 (C-8), 132.7 (C-3), 137.5 (C-6), 138.4 (C-4), 149.7 (C-2), 150.0 (C-8a). IR (KBr, cm^−1^) ν_max_: 3300 (≡C-H), 3282 (≡C-H), 3059 and 2980 (CH_2_, CH_3_), 2129 (C≡C), 1365, 1348, and 1328 (S=O), 1170 and 1143 (S=O). HRMS (ESI) *m*/*z*: C_17_H_16_N_3_NaO_4_S_2_ [M + Na]^+^, Calcd. 413.0480; Found 413.0482.

### 3.6. Procedure for the Synthesis of 6-Chloro-3-quinolinesulfochloride *(**13**)*

#### 3.6.1. Procedure for the Synthesis of 6-Chloro-3-phenylmethylsulfanylquinoline (**10**)

A mixture of S-benzyl-isothiouronium chloride (305 mg, 1.5mmol), NaOH (200 mg, 5 mmol), and dry DMF (10 mL) was heated with stirring at 80 °C under an argon atmosphere for 1 h. Then, 6-chloro-3-bromoquinoline (**7**) (242 mg, 1 mmol) was added, and heating and stirring continued for 6 h. The mixture was cooled to room temperature and poured into ice water (80 mL). The solid was filtered off, washed with water, and air-dried. 6-Chloro-3-phenylmethyl-sulfanylquinoline was recrystallized from a mixture of hexane–CH_2_Cl_2_ (7:3, *v*/*v*) to give pure compound (**10**) (89%). m.p. 77–78 °C (lit. m.p. 77–78 °C [34]).

#### 3.6.2. Chlorination of 6-Chloro-3-phenylmethylsulfanylquinoline (**10**)

A mixture of 10% aqueous solution hydrochloric acid (10 mL), chloroform (10 mL), and 6-chloro-3-phenylmethylsulfanylquinoline **10** (286 mg, 1 mmol) was cooled to 5 °C and then dropped over 60 min to a well-stirred mixture of 6% aqueous solution of sodium hypochlorite (9.5 mL, 6.6 mmol) at such a rate that the temperature remained below 10 °C. The mixture was poured onto 15 g of ice. The organic layer was separated, and the aqueous layer was extracted with chlorform (3 × 3 mL). The chloroform extracts were combined, washed with water, and dried over anhdrous sodium sulfate. CHCl_3_ was evaporated to leave a solid residue. The residue was recrystallized from benzene to give 6-chloro-3-chlorosulfonylquinoline (**13**) (91%). m.p. 109–110 °C (lit. m.p. 109–110 °C [34]).

### 3.7. Biological Study

The cell lines (MCF-7, MDA-MB-231, and nontumor HFF-1) were cultured and kept in Dubecco’s modified Eagle medium (DMEM) supplemented with 10% fetal bovine serum (FBS) 1.000 IU·mL^−1^ penicillin and 10 mg·m^−1^·mL^−1^ streptomycin in atmosphere with 5% CO_2_ at 37 °C. The cells were seeded using a series of standard 96-well plates (Corning). Test compounds were prepared at a concentration of 5.0 mM dimethyl sulfoxide (DMSO). Freshly prepared dilutions of the test compounds in a culture medium at a concentration of 0.5–250 µM were added to the microtiter plates and the cells were grown for 3–4 days. Solvent control (DMSO) was included to check its potential inhibitory activity at the concentration used. After incubation, the cell growth rate was evaluated by performing a WST-1 assay. The amount of formazan produced was measured at 450 nm. Expermentally determined absorbance values were transformed into the cell percentage of growth as described previously. Each test point was performed in triplicate for each tested compound cocentration. The results were statistically analyzed using PraphPad Prism 6 software (Graphpad Software, Inc., La Jolla, CA, USA).

### 3.8. Computational Details

The three-dimensional (3D) structures of all studied sulfamoylquinolines required for virtual screening were generated in their low-energy conformation using a multi-objective genetic algrithm (GA) [35], implemented in the Balloon software [36]. The atomic coordinates of two human cytochrome P450 isozymes (CYPs) were obtained from the PDB [37] using the following PDB keys: 4I8V (CYP1A1) [38] and 3PM0 (CYP1B1) [39]. Computational experiments were carried out using the CLC Drug Discovery Workbench software [40]. The active sites of CYPs were predicted with a resolution of 0.8 Å by the 3D grid-based Find Binding Pockets algorithm. Molecular docking was performed using the search algorithm and the Piecewise Linear Potential Protein-Ligand ANT Sytem (PLANTS_PLP_) empirical scoring algorithm [41]. Ligands were flexible during docking and the chemistry of CYPs was restored to the physiological conditions. For each compound, 100 iterations were executed in the active site of CYP1A1 and CYP1B1, but only the binding mode with the lowest potential energy was selected as a docking result for each CYP-compound complex. Molecular docking details were visualized using the BIOVIA Discovery Studio virtual environment [42]. The atomic distances of ligands situated very close to the ferrous ion (Fe^2+^) of heme in active sites of CYPs were calculated using the Molegro Molecular Viewer (MMV) [43].

## 4. Conclusions

In conclusion, the design, synthesis, structure, and in vitro antiproliferative activity of new quinolinesulfonamide derivatives were presented in this paper. Compounds were prepared using a straightforward and efficient method of synthesis. The molecular structures of the title compounds were confirmed by ^1^H-, ^13^C-NMR, IR, and HR MS spectra. The in vitro anti-breast cancer activity of the synthesized compounds was tested in MCF-7 (human adenocarcinoma), MDA-MB-231, and T-47D (human ductal carcinoma) cells. It is noteworthy that most of the compounds exhibited higher anticancer activity than the reference drug cisplatin. The docking results are consistent with the cytotoxicity assays. The potent acetylenic quinolinesulfonamides against the MDA-MB-231 and MCF-7 cell lines, 2-(3-propynylthio)-6-[*N*-methyl-*N*-(3-propynyl)- sulfamoyl]quinoline (**5b**) and 2-(3-propynyl-seleno)-6-[*N*-methyl-*N*-(3-propynyl)sulfamoyl]- quinoline (**6b**), were ranked highly by the PLANTS_PLP_ scoring function for CYP1A1 and CYP1B1. The quinoline moiety of acetylenic quinolinesulfonamides form a number of hydrophobic interactions with phenylalanines of CYP1A1 (Phe123, Phe224, Phe258) and CYP1B1 (Phe134, Phe231, Phe268). The key to understanding the differences between the binding mechanisms of selected compounds is the orientation of the sulfamoyl and/or thiopropargyl groups inside CYP catalytic sites, very close to the heme–iron center. The superposition of acetylenic quinolinesulfonamide functional groups is stabilized by essential hydrophobic interactions with Ile115, Leu312, Ala317, Val382, Leu386, and Leu490 (CYP1A1) and Val 126, Ala133, Leu264, Ala330, Val395, Ile399, and Leu509 (CYP1B1). The orientation of sufur-containing substituents (sulfamoyl and/or thiopropargyl groups) inside active sites can be additionally enhanced by π–sulfur miscellaneous interactions, mainly with Phe123 or Phe224 (CYP1A1) and Phe134 or Phe268 (CYP1B1) residues. Docking data remain in good correlation with cytotoxic activity results.

This observation suggests that, although the WST-1 screening protocol did not decide on a mechanism for the observed cytotoxic activity of the tested compounds, the activity of **5b** and **6b** may be attributed to the inhibition of the CYP routes. These findings can be exploited in favor of the development and testing of novel quinoline-based molecules for better anticancer activity.

## Figures and Tables

**Table 1:** Cytotoxic activity of acetylenic quinolines and cisplatin as a reference compound.

Compound	Human Cell Line/IC_50_ ± SD [µM]			
	MCF-7	T47D	MDA-MB-231	HFF-1
**2a**	351.36 ± 6.32	neg ^1^	139.82 ± 1.98	neg
**2b**	22.89 ± 1.45	0.30 ± 0.03	0.24 ± 0.01	neg
**2c**	0.17 ± 0.02	2.24 ± 0.11	0.27 ± 0.02	85.37 ± 1.60
**2d**	233.84 ± 1.56	292.04 ± 2.34	180.27 ± 2.35	425.17 ± 1.46
**2e**	1.29 ± 0.09	26.80 ± 0.67	59.31 ± 1.56	neg
**2f**	61.22 ± 1.65	295.64 ± 2.56	204.08 ± 3.10	153.40 ± 3.0
**5a**	287.87 ± 3.45	neg	11.75 ± 0.87	neg
**5b**	18.36 ± 0.98	181.81 ± 3.23	0.27 ± 0.03	neg
**5c**	neg	205.36 ± 6.02	116.15 ± 2.13	neg
**5d**	236.81 ± 2.34	neg	202.00 ± 5.46	neg
**5e**	0.27 ± 0.03	0.21 ± 0.01	7.12 ± 0.78	276.06 ± 2.60
**5f**	13.09 ± 0.11	0.27 ± 0.03	21.21 ± 0.96	366.06 ± 1.46
**6a**	237.93 ± 2.11	146.26 ± 1.98	0.42 ± 0.01	neg
**6b**	22.72 ± 0.12	10.58 ± 0.15	1.32 ± 0.12	92.85 ± 2.0
**6c**	112.67	313.48 ± 7.98	17.56 ± 0.87	neg
**6d**	259.25 ± 5.45	neg	0.68 ± 0.09	330.95 ± 4.0
**6e**	108.75 ± 2.11	159.86 ± 3.23	23.46 ± 1.02	neg
**6f**	0.26 ± 0.01	55.63 ± 1.78	64.47 ± 1.98	317.46 ± 5.46
**11**	7.36 ± 0.45	126.84 ± 2.01	8.73 ± 0.78	175.83 ± 3.0
**12**	3.14 ± 0.34	2.50 ± 0.09	2.86 ± 0.34	375.47 ± 3.0
**14**	118.29 ± 2.04	11.73 ± 1.11	26.83 ± 1.45	71.76 ± 1.60
Cisplatin	77.76 ± 0.76	30.26 ± 0.34	17.63 ± 0.31	7.2 ± 0.60

^1^ Negative in concentration used.

**Table 2:** The results of docking acetylenic sulfamoylquinolines to the active sites of CYP1A1 (PDB ID: 4I8V) and CYP1B1 (PDB ID: 3PM0).

Compound			Interaction Energy (Score) between the Ligand and CYPs (Arbitrary Unit) *							
			Active Site of CYP1A1 (Volume 250.37 Å^3^)				Active Site of CYP1B1 (Volume 214.53 Å^3^)			
No.	Weight (Da)	Flexible Bonds	Total	H-Bond	Steric	Ligand Penalty	Total	H-Bond	Steric	Ligand Penalty
**2a**	294.75	4	−69.57	0.00	−71.29	1.72	−70.02	0.00	−72.00	1.97
**2b**	294.75	4	−73.39	0.00	−74.40	1.00	−71.11	0.00	−72.12	1.01
**2c**	294.75	4	−68.07	0.00	−69.29	1.22	−71.88	0.00	−73.41	1.53
**2d**	294.75	4	−70.40	0.00	−71.62	1.22	−74.76	0.00	−76.36	1.61
**2e**	294.75	4	−70.12	0.00	−71.13	1.02	−72.70	0.00	−73.80	1.10
**2f**	294.75	4	−72.18	0.00	−73.89	1.72	−74.12	0.00	−75.80	1.68
**5a**	330.42	7	−73.22	0.00	−76.14	2.92	−70.11	0.00	−72.24	2.14
**5b**	330.42	7	−81.05	0.00	−84.89	3.83	−76.45	0.00	−80.29	3.84
**5c**	330.42	7	−77.64	0.00	−80.96	3.32	−73.07	0.00	−76.84	3.77
**5d**	330.42	7	−76.83	0.00	−80.24	3.40	−71.88	0.00	−75.08	3.20
**5e**	330.42	7	−76.49	−1.59	−78.25	3.36	−74.32	0.00	−77.37	3.05
**5f**	330.42	7	−75.33	0.00	−78.49	3.16	−76.95	0.00	−79.79	2.84
**6a**	377.32	7	−72.57	0.00	−80.45	7.87	−65.20	0.00	−73.18	7.98
**6b**	377.32	7	−79.23	0.00	−81.22	1.99	−81.55	0.00	−83.49	1.93
**6c**	377.32	7	−80.72	0.00	−83.19	2.47	−70.84	0.00	−72.35	1.50
**6d**	377.32	7	−79.98	0.00	−82.45	2.47	−83.69	0.00	−85.93	2.25
**6e**	377.32	7	−72.09	−2.00	−71.53	1.44	−73.53	0.00	−76.02	2.49
**6f**	377.32	7	−79.11	0.00	−81.49	2.38	−80.21	0.00	−82.29	2.08
**11**	269.38	6	−80.80	−1.88	−81.63	2.71	−76.50	0.00	−78.79	2.29
**12**	391.46	8	−77.86	0.00	−81.48	3.61	−78.01	0.00	−80.19	2.18
**13**	294.75	4	−68.83	0.00	−70.68	1.86	−73.50	0.00	−74.31	0.81

* The distance between protein atoms and ligand atoms is less than 5.5 Å.

## Supplementary Materials

Supplementary materials are available online.
