# Supplementary material for: Synthesis, Anti-Breast Cancer Activity, and Molecular Docking Study of a New Group of Acetylenic Quinolinesulfonamide Derivatives
Source: Molecules. 2017 Feb 16;22(2):300. doi: 10.3390/molecules22020300 (PMC6155812; doi:10.3390/molecules22020300)
Supplement: Supplementary file 1 [file molecules-22-00300-s001.pdf]

Table S1. The distances between docked acetylenic sulfamoylquinoline derivatives and heme-iron center in active sites of CYP1A1 (PDB ID: 4I8V) and CYP1B1 (PDB ID: 3PM0).

Compound	CYP1A1		CYP1B1	
	Ligand Group	Closest Atom Distance to Fe (Å)	Ligand Group	Closest Atom Distance to Fe (Å)
**2a**	sulfamoyl	3.34	sulfamoyl	3.11
**2b**	sulfamoyl	3.18	sulfamoyl	3.52
**2c**	sulfamoyl	3.50	sulfamoyl	3.50
**2d**	sulfamoyl	3.61	sulfamoyl	3.53
**2e**	sulfamoyl	3.29	sulfamoyl	3.30
**2f**	sulfamoyl	3.02	sulfamoyl	3.46
**5a**	sulfamoyl	3.58	sulfamoyl	4.02
**5b**	thiopropargyl	3.64	thiopropargyl	3.94
**5c**	sulfamoyl	3.15	sulfamoyl	3.43
**5d**	sulfamoyl	4.43	sulfamoyl	3.36
**5e**	thiopropargyl	4.33	thiopropargyl	4.28
**5f**	thiopropargyl	3.19	sulfamoyl	3.56
**6a**	selenopropargyl	4.18	sulfamoyl	4.10
**6b**	sulfamoyl	3.36	sulfamoyl	3.48
**6c**	sulfamoyl	3.22	sulfamoyl	4.10
**6d**	sulfamoyl	3.05	sulfamoyl	3.41
**6e**	sulfamoyl	4.02	sulfamoyl	3.49
**6f**	sulfamoyl	2.92	sulfamoyl	3.48
**11**	quinoline moiety	3.68	thiopropargyl	4.33
**12**	sulfamoyl	3.01	sulfamoyl	3.39
**13**	sulfamoyl	3.38	sulfamoyl	3.54

Table S2. Amino acid residues of CYP1A1 (PDB ID: 4I8V) which formed a favorable interactions witch acetylenic sulfamoylquinolines during docking simulation.

Ligand	Hydrophobic interactions						Hydrogen bonds		Others	
	π–π Stacked	π–π T-shaped	Amide–π Stacked	π–Alkyl	π–Sigma	Alkyl	Classic	Carbon	π–Sulfur	Halogen
**2a**	Phe224(2x)	no contact	Leu312/Asp313, Gly316/Ala317	Phe224, Ala317	no contact	Val382, Leu496	no contact	no contact	Phe123	no contact
**2b**	Phe224(2x)	no contact	Gly316/Ala317(2x)	Phe224, Phe258, Ala317(2x), Phe319	no contact	Val382, Le386, Leu496	no contact	no contact	no contact	no contact
**2c**	Phe224(2x)	no contact	Gly316/Ala317(2x)	Phe224, Leu312, Ala317	Gly316	Val382, Leu496	no contact	Ala317	Phe123	no contact
**2d**	Phe224(2x)	Phe258	Gly316/Ala317(2x)	Phe224, Leu312, Ala317, Phe319	Gly316	Val382, Leu496	no contact	Asp313, Ala317	Phe123	no contact
**2e**	Phe224(2x)	no contact	Gly316/Ala317(2x)	Phe224, Ala317(2x), Phe319	no contact	Val382, Le386, Leu496	no contact	Ala317	no contact	no contact
**2f**	Phe224(2x)	no contact	Gly316/Ala317(2x)	Phe224, Ala317(2x)	Phe123	Val382, Leu496	no contact	no contact	no contact	no contact
**5a**	Phe224(2x)	no contact	Gly316/Ala317(2x)	Ala317	no contact	Leu312, Val382, Leu496	no contact	Ser122	Phe123	no contact
**5b**	Phe224	Phe123(2x)	Gly316/Ala317	Phe224, Phe258, Ala317, Phe319	Phe123, Ala317	Val382, Leu496	no contact	Leu312	no contact	no contact
**5c**	Phe224(2x)	no contact	Gly316/Ala317(2x)	Ala317	no contact	Ile115, Leu312, Val382, Ile386, Leu496	no contact	no contact	Phe123	no contact
**5d**	Phe224(2x)	Phe258	Gly316/Ala317(2x)	Phe123	no contact	Ala317, Val382, Ile386, Leu496	no contact	Ala317	Phe123	no contact
**5e**	Phe224(2x)	Phe123(2x)	Gly316/Ala317	Phe224, Phe258, Ala317, Phe319	Phe123, Ala317	Val382	no contact	Leu312	no contact	no contact
**5f**	Phe224	Phe123(2x)	Gly316/Ala317	Phe258, Ala317	Ala317	Leu312, Val382	no contact	no contact	Phe123	no contact
**6a**	Phe224(2x)	Phe258	Gly316/Ala317	Phe123, Ala317	no contact	Ile115, Val382, Leu496	Thr321	no contact	no contact	no contact
**6b**	Phe224(2x)	no contact	Gly316/Ala317(2x)	Phe224, Phe258, Ala317, Phe319	no contact	Val382, Leu496	no contact	no contact	Phe123	no contact
**6c**	Phe224	Phe123(2x)	Gly316/Ala317(2x)	Phe224, Ala317	Phe123, Ala317	Val382, Leu496(2x)	no contact	no contact	no contact	no contact
**6d**	Phe224(2x)	no contact	Gly316/Ala317(2x)	Phe258, Ala317	no contact	Leu312, Val382, Leu496	no contact	no contact	no contact	no contact
**6e**	Phe224(2x)	Phe123	Gly316/Ala317	Ile115, Phe224, Leu312, Ala317, Phe319	Phe123	Leu496	Ser122	no contact	no contact	no contact
**6f**	Phe224(2x)	no contact	Gly316/Ala317(2x)	Phe258, Ala317(2x)	no contact	Leu312, Val382	no contact	no contact	no contact	no contact
**11**	Phe224	Phe123(2x)	Gly316/Ala317	Phe224, Phe258, Ala317, Phe319	Phe123, Ala317	Leu254, Val382	Ser122	no contact	Phe224	no contact
**12**	Phe224(2x)	no contact	Gly316/Ala317(2x)	Phe123, Ala317	no contact	Ile115, Val382	no contact	Gly316, Leu312(2x), Asn225	Phe258	no contact
**13**	Phe224(2x)	Phe123	Gly316/Ala317(2x)	Phe224, Phe258, Ala317	Ala317	Val382	no contact	no contact	no contact	no contact

Table S3. Amino acid residues of CYP1A1 (PDB ID: 3PM0) which formed a favorable interactions witch acetylenic sulfamoylquinolines during docking simulation.

Ligand	Hydrophobic interactions						Hydrogen bonds		Others	
	π–π Stacked	π–π T-Shaped	Amide–π STACKED	π–Alkyl	π–Sigma	Alkyl	Classic	Carbon	π–Sulfur	Halogen
**2a**	Phe231(2x)	no contact	Gly329/Ala330(2x)	Phe231, Ala330(2x)	no contact	Val395, Ile399, Leu509(2x)	no contact	no contact	no contact	Asp333
**2b**	Phe231(2x)	Phe134	Gly329/Ala330(2x)	Ala133, Phe268	Ala330	Val395, Leu509	no contact	no contact	no contact	no contact
**2c**	Phe231(2x)	no contact	Gly329/Ala330(2x)	Phe231, Ala330	no contact	Val395, Leu509	no contact	Asp326, Ala330	Phe134	Asp333
**2d**	Phe231(2x)	no contact	Gly329/Ala330(2x)	Phe231, Ala330	no contact	Val395, Leu509	no contact	no contact	Phe134	no contact
**2e**	Phe231(2x)	no contact	Gly329/Ala330(2x)	Phe231, Ala330(2x)	no contact	Val395, Ile399, Leu509	no contact	no contact	no contact	no contact
**2f**	Phe231(2x)	no contact	Gly329/Ala330(2x)	Phe231, Phe268, Ala330(2x)	no contact	Val395, Leu509	no contact	no contact	Phe134	no contact
**5a**	Phe231(2x)	Phe268	Gly329/Ala330(2x)	Ala330	no contact	Val126, Val395, Leu509	no contact	Gln332, Asp333	Phe134(2x)	no contact
**5b**	Phe231	Phe134(2x)	Gly329/Ala330(2x)	Ala133, Phe231, Ala330	Ala330	Val395, Leu509	no contact	Asn265, Gly329	no contact	no contact
**5c**	Phe231(2x)	no contact	Gly329/Ala330(2x)	Ala330	no contact	Val126, Val395, Ile399, Leu509	no contact	no contact	Phe134	no contact
**5d**	Phe231	Phe134	Gly329/Ala330(2x)	Ala133, Phe231, Phe268, Ala330	Ala330	Leu264, Val395, Leu509	no contact	Asp326, Ala330, Asp333	Phe134	no contact
**5e**	Phe231	Phe134	Gly329/Ala330(2x)	Ala133, Phe231, Ala330	Ala330	Val395, Leu509	no contact	Asn265, Gly329	Phe134	no contact
**5f**	Phe231(2x)	no contact	Gly329/Ala330(2x)	Ala330(2x)	no contact	Val126, Val395, Ile399, Leu509	no contact	Asp326	Phe134, Phe268	no contact
**6a**	Phe231(2x)	Phe268	Gly329/Ala330(2x)	Ala330(2x)	no contact	Ala133, Ala330, Val395, Leu509	no contact	Gln332, Asp333	Phe134(2x)	no contact
**6b**	Phe231(2x)	no contact	Gly329/Ala330(2x)	Val126, Phe231, Ala330	no contact	Leu264, Val395, Ile399, Leu509	no contact	no contact	Phe134	no contact
**6c**	Phe231(2x)	Phe268	Gly329/Ala330(2x)	Ala330	no contact	Ala133, Ala330, Val395, Leu509	no contact	no contact	Phe134	no contact
**6d**	Phe231(2x)	no contact	Gly329/Ala330(2x)	Phe231, Phe268, Ala330	no contact	Leu264, Val395, Leu509	no contact	Asp326	Phe134	no contact
**6e**	Phe231(2x)	no contact	Gly329/Ala330(2x)	Val126, Phe231, Ala330	no contact	Ala133, Leu264, Ala330	no contact	Asn228, Asp326	Phe134	no contact
**6f**	Phe231(2x)	no contact	Gly329/Ala330(2x)	Ala133, Phe231, Phe268, Ala330(2x)	no contact	Leu264, Val395, Leu509	no contact	no contact	Phe134	no contact
**11**	no contact	Phe134	Gly329/Ala330	Ala133(2x), Phe231, Phe268	Ala330(2x)	Val395, Ile399, Leu509	no contact	Asp326	Phe134	no contact
**12**	Phe231(2x)	Phe134(2x)	Gly329/Ala330	Ala133, Phe231, Ala330	Ala330	Val395, Ile399, Leu509	no contact	Asn265, Asp326(2x), Gly329	no contact	no contact
**13**	Phe231	Phe134	Gly329/Ala330(2x)	Ala133, Phe231, Phe268	Ala330	Leu264, Val395, Leu509	no contact	Asp326	no contact	no contact
